# First Serologic Evidence of West Nile Virus and Usutu Virus Circulation Among Dogs in the Bulgarian Danube Region and Analysis of Some Risk Factors

**DOI:** 10.3390/vetsci12040373

**Published:** 2025-04-16

**Authors:** Nikolina Rusenova, Anton Rusenov

**Affiliations:** 1 Department of Veterinary Microbiology, Infectious and Parasitic Diseases, Faculty of Veterinary Medicine, Trakia University, 6000 Stara Zagora, Bulgaria; 2 Department of Internal Diseases, Faculty of Veterinary Medicine, Trakia University, 6000 Stara Zagora, Bulgaria; anton.rusenov@trakia-uni.bg

**Keywords:** West Nile virus, Usutu virus, seroprevalence, ELISA, VNT, dogs, Danube region, Bulgaria

## Abstract

This study aimed to assess West Nile virus (WNV) and Usutu virus seroprevalence among the dog population in the Danube region, Bulgaria, to confirm the results of ELISA by the virus neutralisation test (VNT), as well as to analyse several risk factors of seropositivity in dogs. To implement this, a total of 201 blood samples were collected from dogs in four districts bordering the Danube River. All the samples were tested for anti-WNV protein E antibodies using competitive ELISA. Neutralising antibodies against WNV and Usutu virus were tested in all the ELISA-positive samples. The results show a WNV seroprevalence of 45.3% (*n* = 91, CI = 36.45–55.59) by ELISA, whereas the virus neutralisation test indicated a seroprevalence of 21.9% (n = 44, CI = 15.91–29.39). Neutralising antibodies against Usutu virus were detected for the first time in Bulgaria, with a prevalence of 6% (n = 12, CI = 3.09–10.43). Compared to VNT, ELISA demonstrated 100.0% sensitivity and 70.1% specificity. The region (*p* < 0.0187), the district (*p* = 0.0258) and the ages of the dogs (*p* = 0.0180) were identified as statistically significant risk factors associated with WNV seropositivity. This study provides indirect evidence of WNV and Usutu virus circulation among dogs in the Danube region of Bulgaria, highlighting a potential risk for susceptible hosts in the area.

## Simple Summary

West Nile virus (WNV) and Usutu virus are arboviruses primarily transmitted by mosquitoes. This study aimed to assess the seroprevalence of these viruses among the dog population in the Danube region of Bulgaria to confirm the results of ELISA against the reference method—virus neutralisation test (VNT)—and to analyse some risk factors associated with seropositivity in dogs. Two hundred one blood serum samples were collected from dogs in four districts in Bulgaria bordering the Danube River. Overall, 44 samples were confirmed positive for WNV, resulting in a seroprevalence of 21.9%. Antibodies against Usutu virus were detected for the first time in Bulgaria, with a prevalence of 6%. The region, the district and the dogs’ ages were risk factors associated with WNV seropositivity. This study demonstrates that WNV and Usutu viruses circulate among dogs in the Bulgarian Danube region and highlights a potential risk for susceptible hosts in the area.

## 1. Introduction

The West Nile virus (WNV) is classified under the *Orthoflavivirus* genus in the *Flaviviridae* family [1] and possesses a positive-sense RNA genome that is infectious [2]. It is a member of the arbovirus group, primarily transmitted by mosquito vectors and less frequently by ticks [3]. *Culex* spp. mosquitoes play a key role in maintaining the WNV life cycle on one hand, and wild birds, particularly corvids (crows and jays), passerines and raptors, serve as reservoirs of the virus in nature on the other [4]. Some bird species are also known as amplifiers because they develop high levels of viraemia, which facilitates the infection of vectors during their blood meals [5]. After viral replication in mosquitoes, these bridge vectors can transmit WNV to susceptible hosts, including humans and other mammals [4,6]. Humans, horses, dogs and other vertebrates are, however, considered accidental dead-end hosts because they do not contribute to the transmission cycle of WNV [7,8]. It is well-documented in the literature that approximately 80% of humans infected with WNV remain asymptomatic, and less than 1% of those who develop clinical symptoms progress to neurologic disease [9,10]. Apart from fever, around 10% of horses infected with WNV develop symptoms affecting the central nervous system [11,12]. Dogs are significantly more resistant to disease than horses and humans, and experimental studies have revealed short-lived viraemia with few or no clinical signs [13]. However, sporadic clinical cases of encephalitis [14,15] and myocarditis [14] have also been recorded.

Usutu virus (USUV) is a flavivirus that frequently co-circulates with the West Nile virus, as both share a similar enzootic transmission cycle between birds and ornithophilic mosquitoes [16]. As in the case with WNV, Usutu virus infections in humans and mammals are asymptomatic in the majority of cases [17]. However, it has been documented as a cause of fever, jaundice, skin rash and neuroinvasive disease in humans across several European countries. Antibodies to Usutu virus have been established in various species, including birds, bats, squirrels, wild boars, deer and horses [18].

Horses and dogs have been subjects of extensive studies on the seroprevalence of WNV around the globe as potential sentinels for WNV monitoring and the prediction of human cases [19,20,21,22,23,24,25,26,27,28,29]. For this purpose, the WOAH recommends immunoenzymatic assays, such as ELISA and neutralisation tests [30], although immunofluorescence assays (IFA) and haemagglutination-inhibition tests are also used in laboratories for analysis of a small number of samples [31]. A disadvantage of ELISA formats is the cross-reactivity of antibodies induced by other flaviviruses with both native and recombinant WNV antigens [31,32,33]. WNV and Usutu virus are members of the Japanese encephalitis (JE) serocomplex, together with Kunjin virus (KUNV), Murray Valley encephalitis virus (MVEV), Kokobera virus, Japanese encephalitis virus (JEV), Alfuy virus and St. Louis encephalitis virus (SLEV) [34]. All these viruses are antigenically closely related, but they also share common antigenic determinants with representatives of other flavivirus serocomplexes, such as the tick-borne encephalitis virus [35,36] or Bagaza virus [37]. Cross-reactivity impairs the accuracy of diagnosis and requires more specific tests, such as virus neutralisation, for confirmation [38].

In Bulgaria, the first case of neuroinvasive disease caused by WNV in a human was recorded in 2015 [39]. The nationwide seroprevalence studies conducted in humans in all 28 districts in Bulgaria in 2015 and 2018 revealed a seroprevalence of 1.5% [40] and 1.2%, respectively [41]. In a study involving samples from *Culex* spp. mosquitoes, Christova et al. [42] isolated WNV belonging to the Hungarian clade of WNV lineage 2. In another research study, antibodies to WNV were detected in 17.5% of 160 wild birds from 18 species sampled from two regions of Bulgaria [43]. Serological investigations of sentinel animals for flavivirus circulation in Bulgaria remain scarce. Rusenova et al. [44] reported a WNV seroprevalence of 1% in samples from donkeys and mules collected in 2015. In a more recent survey of equine serum samples from 2022, the seroprevalence was 3.97% [45].

To date, no serological studies on the WNV infection status of dogs have been conducted in Bulgaria. Therefore, the present study aimed to assess the seroprevalence of WNV and Usutu virus in dogs from the Danube region of Bulgaria, as well as to analyse certain factors linked to seropositivity.

## 2. Materials and Methods

### 2.1. Experimental Design and Collection of Samples

**Figure 1:**
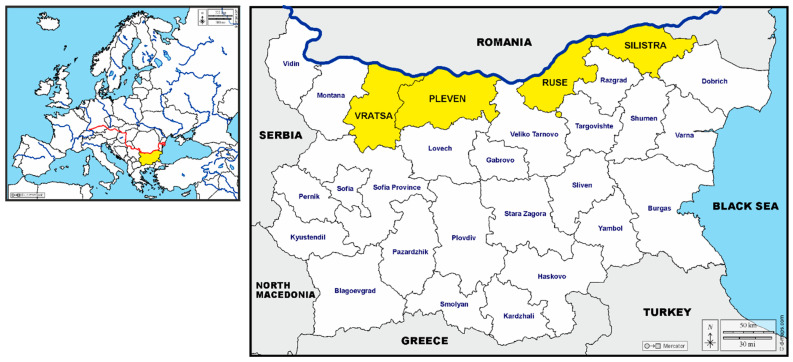
Map showing geographical location of Bulgaria in Europe and the tested districts, with the Danube River as the north boundary.

This study encompasses four districts bordering the Danube River, which is the natural boundary between Bulgaria and Romania (Figure 1). The surveyed areas are characterised by a temperate continental climate, with cold winters and hot summers.

A total of 201 blood samples were collected from dogs either housed in shelters or privately owned, all with permanent outdoor access. The distribution of dogs and corresponding samples by district was as follows: Vratsa, n = 36; Pleven, n = 70; Ruse, n = 50; and Silistra, n = 45. Vratsa and Pleven fall within the north-western region of Bulgaria, Ruse in the north-central, and Silistra in the north-eastern region.

The samples were collected with the consent and in the presence of the owners and/or the veterinarians responsible for the animals, adhering to all humane treatment standards. All the dogs were clinically healthy, with no history of infectious diseases or relocation from other districts two years prior to sampling. Additionally, data regarding the age, sex and breed of the animals were recorded.

Blood samples (2 mL) were obtained using vacutainers with clot activator and 20G needles via puncture of the vena cephalica antebrachii under aseptic conditions. The tubes were stored at 4 °C and processed by centrifugation at 2000× *g* for 10 min within 24 h after collection. The sera were then transferred into Eppendorf tubes and stored at −80 °C until analysis.

### 2.2. Serological Assays

All the serum samples were tested for antibodies specific to glycoprotein E of the West Nile virus using a commercial ELISA kit (Ingezim West Nile Compac, Ingenasa, Madrid, Spain). The kit enables the detection of antibodies from various animal species, including humans, and is based on competition between the antibodies in the serum samples and the monoclonal antibody in the conjugate. Prior to testing, the serum samples underwent heat treatment to eliminate endogenous thermolabile viral inhibitors. The testing procedure strictly followed the manufacturer’s instructions. The optical density (OD) was measured at 450 nm using an ELISA reader (Biosan, Latvia) immediately following addition of the stop solution. The test results were confirmed using the OD values of the negative control (2 wells) (> 0.8) and the positive control (2 wells) (<0.35). For result interpretation, the inhibition percentage (IP) values were considered as follows: IP ≥ 40%—presence of specific anti-WNV antibodies (positive); IP ≤ 30%—absence of specific anti-WNV antibodies (negative); sera with IP from 30% to 40% were considered inconclusive.

Positive, inconclusive and selected negative sera identified using the competitive ELISA (cELISA) Ingezim West Nile Compac were further tested with the cELISA for multiple species produced by IDvet (Grabels, France), and the results were confirmed by a microneutralisation test (VNT). Both additional tests were conducted at the World Organisation for Animal Health (WOAH) Reference Laboratory for WNV in Teramo, Italy.

The competitive ELISA kit (IDvet) also detects antibodies against the envelope protein (prE) of WNV in sera from various biological species based on competition with an enzyme-labelled monoclonal antibody. The results were considered valid if the mean value of the negative control OD (OD_NC_) was greater than 0.700 and the mean value of the positive control was less than 30% of the OD_NC_. The results were interpreted by calculating the S/N percentage as follows: S/N% ≤ 40%—samples were reported as positive; S/N% > 40% and ≤50%—samples were reported as doubtful; S/N% > 50%—samples were considered negative.

The virus neutralisation test (VNT) was performed to detect neutralising antibodies against both WNV and USUV. The test was carried out in 96-well plates, as previously described [46]. The strains Eg101 and 939/01 were used as antigens for West Nile virus and Usutu virus, respectively. Titres were interpreted as the reciprocal of the highest dilution of the serum sample that exhibited no cytopathic effect on Vero cells (100% neutralisation). Each plate included both positive and negative control sera. Titres equal to or greater than 1:10 were considered positive for the respective virus.

If a serum sample had antibodies against both viruses, it was considered positive for the virus whose titre was at least fourfold higher (≥4×) than that of the other virus. If the titres for the two viruses were equal or the difference was less than 4×, the samples were classified as doubtful, indicating no specific immune response [47]. Samples testing positive by competitive ELISA but negative by VNT were considered seronegative.

### 2.3. Statistical Analysis

The age of the sampled dogs was reported as the median and the interquartile range, and the differences between seropositive and seronegative dogs were tested with the Mann–Whitney U-test. The Chi-square test was used to evaluate the differences between seropositive and seronegative dogs for categorical variables (region, district and sex). A non-parametric receiver operating characteristic (ROC) curve analysis using the algorithm of DeLong et al. [48] was applied to calculate the optimal cutoff values of age on the basis of the Youden J statistic. The assessment of the cELISA diagnostic value for WNV diagnosis vs. the “gold assay” VNT was conducted through analysis of the area under the ROC curve (AUCs) as a measure of diagnostic parameter accuracy, test sensitivity and specificity. The AUC interpretation as measure of test’s diagnostic accuracy was done according to criteria listed by Nahm [49]. The analyses were carried out using MedCalc 15.8 statistical software (Belgium).

## 3. Results

A total of 201 samples were collected from dogs, comprising 80 male and 121 female animals aged between 6 months (n = 13) and 13 years (4.6 ± 2.6, mean ± SD). All the animals were of mixed breed. Detailed data on the distribution of samples by sex and age of the animals, as well as the percentage of samples examined by district, are presented in Supplementary Table S1.

Competitive ELISA detected antibodies in 91 out of 201 samples (45.3% [95% CI = 36.45–55.59]), with the regional distribution as follows: Vratsa—16/36 (44.4% [CI = 25.4–72.17]); Pleven—40/70 (57.14% [CI = 40.82–77.81]); Ruse—17/50 (34.0% [CI = 19.81–54.44]); and Silistra—18/45 (40.0% [CI = 23.71–63.22]) (Table 1). One sample classified as doubtful by cELISA in our laboratory was cELISA-positive in the reference laboratory. This sample was subsequently recorded as positive in the cELISA analysis.

Following the VNT analysis, the seroprevalence of WNV was definitively determined to be 21.9% (n = 44 [CI = 15.91–29.39]). Neutralising antibodies were detected in all four investigated districts, ranging from 10.0% in Ruse to 27.8% in the Vratsa district (Table 2).

Neutralising antibodies against Usutu virus were identified in 12 serum samples (6% [CI = 3.09–10.43]) from three districts: Pleven, 8/70 (11.4%); Ruse, 2/50 (4.0%); and Silistra, 2/45 (4.4%) (Table 3).

Doubtful VNT results were identified in nine samples where titres against both tested viruses were either equal (in five dogs) or less than fourfold (in four dogs).

The titres of antibodies against WNV and Usutu viruses, along with their interpretation, are presented in Table 4.

Thirteen dogs exhibited antibodies against WNV with titres of 1:10 (29.5%), 17 with titres of 1:20 (38.6%), 8 with titres of 1:40 (18.2%), 5 with titres of 1:80 (11.4%) and 1 animal with a titre of 1:160 (2.3%). The antibody titres against Usutu virus were as follows: six dogs with titres of 1:10 (50.0%), two with titres of 1:20 (16.7%), three with titres of 1:40 (25.0%) and one animal with a titre of 1:160 (8.3%). In the 26 cELISA-positive sera, no antibodies against WNV and/or Usutu virus were detected; therefore, these samples were classified as having undetermined flavivirus exposure.

**Figure 2:**
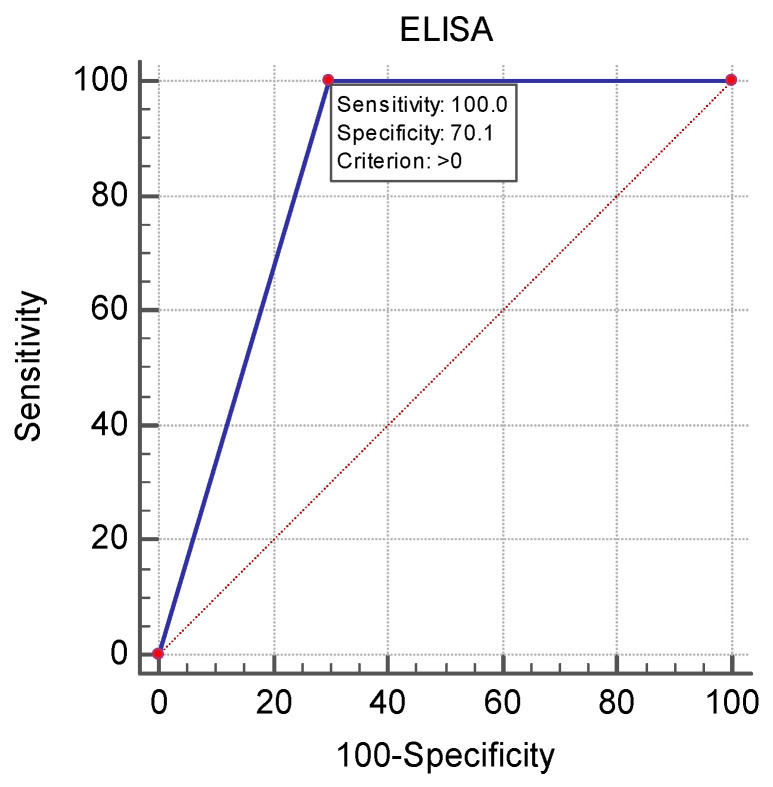
ROC curve analysis for the diagnostic performance of c-ELISA.

The ROC curve analysis of the diagnostic performance of cELISA compared to VNT (Figure 2) demonstrated an AUC = 0.850, with a 95% confidence interval ranging from 0.6242 to 0.7707 (*p* < 0.0001). The cELISA had 100.0% sensitivity and 70.1% specificity.

The data from the logistic regression analysis of WNV seroprevalence and associated independent variables are presented in Table 5.

Dogs raised in the northwestern region of Bulgaria were at three times higher risk to become infected with WNV (*p* = 0.0187) as compared to those in the north-central region. The risk for dogs located in the Ruse district was approximately three times lower than for those raised in the Pleven district (*p* = 0.0258). Finally, dogs aged ≥2 years had a three times higher risk of infection (*p* = 0.0180) than dogs younger than 2 years.

## 4. Discussion

During the past decade, WNV has been identified as a serious public health threat in different European countries, including Balkan neighbouring countries of Bulgaria: Greece, Romania and Serbia [50,51]. Other than in humans, cases have been registered in horses and birds, with 51% more outbreaks among equids in 2023 compared to 2022 [52]. The present study sheds light, although indirectly, on WNV and Usutu virus circulation among the canine population in the Danube region of Bulgaria.

Out of the 201 serum samples tested for anti-WNV IgG antibodies by cELISA, 91 (45.3% [95% CI = 36.45–55.59]) were positive, whereas the virus neutralisation test confirmed 44 of them (21.9% [95% CI = 15.91–29.39]). According to the manufacturer, only WNV-suspect sera are confirmed by VNT. The literature overview, however, demonstrates that in serological tests for detection of flaviviruses, including WNV, IgG-ELISA results are compared to the gold standard—the virus neutralisation test—for accurate interpretation without false positive results [53,54,55].

The results from the tests of ELISA-positive samples with the reference method also demonstrated the presence of neutralising antibodies against the Usutu virus (6% [95% CI = 3.09–10.43]). Nine sera were interpreted by VNT as doubtful because of equal antibody titres with less than four-fold difference. These findings may be explained by the presence of co-infection with both viruses, which does not raise a specific immune response against the tested viruses. The overview of the results from the ELISA-tested sera drew our attention to the association between the samples’ IP and VNT-Usutu seropositivity. Thus, sera positive for anti-Usutu virus neutralising antibodies demonstrated lower IP values from 40.06% and 69.77%. Additionally, the IP values of samples positive for WNV antibodies were over 91.67%, while only one sample had an IP of 88.48%. The ELISA-positive but WNV- or Usutu virus-negative samples had IP values from 78.90% to 93.31%, with the exception of one sample with IP 54.14%. The high number of samples that were not confirmed by VNT motivated us to determine the sensitivity and specificity of ELISA in relation to the gold standard by ROC analysis. The results show a good diagnostic value of the test with AUC = 0.850, sensitivity 100%, but a relatively moderate specificity of 70.1%. A previous study of ours on the WNV seroprevalence in equine sera reported ELISA specificity of 94.5%, but it did not detect the Usutu virus, and the number of samples from the Danube region was smaller (n = 51) [45]. Commercial WNV ELISA kits may be preferred for screening serosurveys due to their advantages, such as simplicity, ease of use, low cost and no need for biosafety level 3 laboratories [56,57]. However, the region and the circulation of other flaviviruses should also be taken into account [58].

The WNV seroprevalence determined by the VNT varied from 10% in the Ruse district to 27.8% in the Vratsa district, whereas ELISA detected a prevalence between 34% (Ruse district) and 57.1% (Pleven district). The data were comparable to those obtained in equids from the Montana district—VNT-WNV seroprevalence of 28.21% vs. ELISA-WNV seroprevalence of 43.59%. The sampled horses from the Vratsa district (both districts being located along the Danube River in the north) were WNV-seronegative, probably due to their small number (n = 11) and restricted outdoor stay [45]. A study from 2015 including 1451 residents revealed a seroprevalence of 6% for the Ruse and Silistra districts, with detection of neutralising antibodies in 1 out of 50 tested samples (2%) from the Vratsa and Silistra districts. Another Bulgarian serological study in humans conducted in 2018 by ELISA has registered the highest seroprevalence in the Pleven district—4.29%, followed by Silistra (2.86%) and Ruse and Vratsa districts, both with 1.43% [41].

A surprising finding in the present survey was the first detection of neutralising antibodies against the Usutu virus in Bulgaria. Such antibodies were absent in previous studies on a total of 578 samples from odd-hoofed animals in Bulgaria [44,45] and in human sera originating from all districts in the country [40]. Overall, 12 dogs (6%) with antibodies were detected in three out of the four studied districts—Pleven (n = 8), Ruse and Silistra (n = 2 in each). The main vectors of the Usutu virus are *Aedes* genus mosquitoes, which transmit the virus from amplifying reservoirs, such as Eurasian blackbirds (*Turdus merula*) and great grey owls (*Strix nebulosa*) [18], the latter being widely spread in Bulgaria. The first-time detection of Usutu virus antibodies among dogs in Bulgaria underscores the expanding geographic range of the virus in the region. This finding could have implications for future surveillance efforts, as the Usutu virus has been linked to avian populations in Bulgaria, which are of key importance to its transmission dynamics. The presence of antibodies in both dogs and humans, shown in this study and previous research, indicates that these viruses are actively circulating and highlights the importance of monitoring vector populations and wildlife reservoirs. Thus, we can better understand the risks these viruses pose to both animal and human populations in the region.

The circulating neutralising antibodies against West Nile and Usutu viruses detected in the present study indicate that dogs with constant outdoor access are at risk of being bitten by infected mosquitoes, which find favourable conditions for replication in the Danube region wetlands. There are about 70 Bulgarian islands in the Danube River [59], home to various wild bird species, which, together with the vectors, maintain the enzootic transmission cycle of these viral infections. In support of our study’s findings, Trifonova et al. [43] reported antibodies against WNV in five wild bird species caught in the Kalimok region, which is located in the territory of two studied districts—Ruse and Silistra.

The antibody titres against WNV and Usutu viruses established in the present study were between 1:10 and 1:160, with prevailing titres of 1:10–1:20 detected in a total of 38 animals. Most probably, they indicate a past infection, given that in infected subjects, the IgG class antibodies may circulate at low titres for years [60], although the retention time of antibodies in dogs is unrecognised [61]. Class IgM antibodies, evidencing a recent infection, were not tested in this study, which is a limitation.

Attempts for isolation of RNA from blood samples were not made because the tested dogs were clinically healthy, the viraemia in dogs is at a low level and is short-lived and the molecular methods are useful at the onset of the disease. It may be speculated that the animals were exposed to genetic lineage 2 WNV viruses whose circulation was confirmed in the country and the Balkan Peninsula [42,62].

In the present study, there was a relatively high number of sera that reacted positively in the WNV-ELISA kit (n = 26) but tested negative for WNV and Usutu virus and were therefore interpreted as samples with undetermined flaviviruses. These samples originated from all four studied districts, and the results may be attributed to other untested but closely related flaviviruses that possibly circulate in the Danube region. This finding confirms observations from previous studies of ours that identified undetermined antibodies in 20 out of 35 ELISA-WNV positive samples from equids, including samples from the Danube region (Montana district) [45], as well as in 12 out of 14 positive samples from donkeys, including in the Pleven district [44]. The data suggest that humans and animals in the Danube regions may be at risk from infection with other unidentified flaviviruses, such as the tick-borne encephalitis virus (TBEV). TBEV was detected in 0.6% of tested human serum samples in Bulgaria, with the highest seroprevalence found in the Gabrovo (4.8%) and Ruse (4%) districts [40].

Circulation of neutralising antibodies against the WNV and Usutu virus has been confirmed in clinically healthy dogs from other countries in the Danube region and Europe. In Serbia, Vasic et al. [55] tested 184 sera and found 68 positive for WNV and 4 positive for Usutu virus, with highest seropositivity rates in places near two rivers, including the Danube River. In Slovenia, the seroprevalence of WNV in serum samples from dogs in 2017 was 1.4% and was 3.7% in 2018, respectively [63]. In the survey on flavivirus circulation in hunting dogs in the Campania region of Italy, Montagnaro et al. [64] detected 24/183 (13.11%) sera positive for Usutu virus. Laidoudi et al. [47] reported that 25/310 dogs (8.1%) tested positive for WNV and/or USUV in the southeast of France.

Some risk factors that may possibly influence WNV seropositivity were additionally investigated in the present study. The tested variables included the origin of samples by region, by district and the age of sampled dogs. The breed of animals was not included, as all the studied dogs were mixed breed. The dogs from the northwestern region were at higher risk of infection vs. those from the north-central region, and the risk for dogs from the Ruse district was the lowest. In addition, dogs older than 2 years were three times more likely to get infected (*p* = 0.0180) than dogs younger than 2 years. Antibodies were not detected in dogs under one year of age, contrary to the findings of Resnick et al. [65], in which there was about 18% average seroprevalence in juvenile animals (6–28 weeks of age). Having studied the WNV seroprevalence in indoor dogs in Iași county in Romania, Oșlobanu et al. [66] found higher rates in dogs older than 10 years, but the difference was not significant.

## 5. Conclusions

The present study demonstrates the circulation of WNV, Usutu virus and other undetermined flaviviruses among the dog population in the Danube region of Bulgaria. The high WNV seroprevalence rates in the present and in previous studies in equids indicate the infection endemicity in the region, which poses the issue of protecting human and animal health through joint efforts of human and veterinary authorities. Repeated studies of the susceptible population and assessment of the potential of dogs as sentinel animals are necessary. The established low specificity of ELISA kits for WNV in the canine serum samples shows that precise detection can be carried out with VNT to differentiate antibodies induced by flaviviruses from the same or different serocomplexes. Future integral studies, also based on molecular methods, are needed to fully understand the epidemiology of flavivirus infections in the region and the country as a whole.

## Figures and Tables

**Table 1:** Seroprevalence of WNV in tested dogs according to cELISA results.

Parameter		Negative, n = 110 Median (IQR) or Number (%)	Positive, n = 91 Median (IQR) or Number (%)	*p*-Value
Region	Northwestern	50 (47.2)	56 (52.8)	0.0635
	North-central	33 (66.0)	17 (34.0)	
	Northeastern	27 (60.0)	18 (40.0)	
District	Vratsa	20 (55.6)	16 (44.4)	0.0700
	Pleven	30 (42.9)	40 (57.1)	
	Ruse	33 (66.0)	17 (34.0)	
	Silistra	27 (60.0)	18 (40.0)	
Age, years		4 (1–5)	5 (3–6)	0.0073
Sex	Female	63 (52.1)	58 (47.9)	0.4312
	Male	47 (58.7)	33 (41.2)	

**Table 2:** WNV-VNT seroprevalence in 201 serum samples from dogs in Danube region, Bulgaria.

Parameter		Negative, n = 157 Median (IQR) or Number (%)	Positive, n = 44 Median (IQR) or Number (%)	*p*-Value
Region	Northwestern	77 (72.6)	29 (27.4)	0.0500
	North-central	45 (90.0)	5 (10.0)	
	Northeastern	35 (78.8)	10 (22.2)	
District	Vratsa	26 (72.2)	10 (27.8)	0.1118
	Pleven	51 (72.9)	19 (27.1)	
	Ruse	45 (90.0)	5 (10.0)	
	Silistra	35 (78.8)	10 (22.2)	
Age, years		4 (2–6)	4.5 (3.5–6)	0.1129
Sex	Female	93 (76.9)	28 (23.1)	0.7242
	Male	64 (80.0)	16 (20.0)	

**Table 3:** Seroprevalence of Usutu virus in dogs in the studied provinces and regions in Bulgaria determined by VNT.

N Samples	VNT-WNV Titre	VNT-USUV Titre	Interpretation
13	1:10	ND	WNV
16	1:20	ND	WNV
1	1:20	1:5	WNV
7	1:40	ND	WNV
1	1:40	1:10	WNV
3	1:80	ND	WNV
1	1:80	1:10	WNV
1	1:80	1:20	WNV
1	1:160	1:10	WNV
6	ND	1:10	USUV
2	ND	1:20	USUV
2	ND	1:40	USUV
1	1:10	1:40	USUV
1	1:40	1:160	USUV
2	1:10	1:10	WNV + USUV
1	1:10	1:20	WNV + USUV
1	1:20	1:10	WNV + USUV
3	1:20	1:20	WNV + USUV
1	1:40	1:20	WNV + USUV
1	1:40	1:40	WNV + USUV
26	ND	ND	UD *
Total 91			44 WNV, 12 USUV, 9 WNV/USUV, 26 UD

* UD = undetermined flaviviruses.

**Table 4:** Neutralising antibody titres for WNV and Usutu viruses in serum samples from dogs determined in cELISA-positive samples.

Parameter		Negative, n = 189 Median (IQR) or Number (%)	Positive, n = 12 Median (IQR) or Number (%)	*p*-Value
Region	Northwestern	98 (92.5)	8 (7.5)	0.6060
	North-central	48 (96.0)	2 (4.0)	
	Northeastern	43 (95.6)	2 (4.4)	
District	Vratsa	36 (100.0)	-	0.0884
	Pleven	62 (88.6)	8 (11.4)	
	Ruse	48 (96.0)	2 (4.0)	
	Silistra	43 (95.6)	2 (4.4)	
Age, years		4 (2–6)	4.5 (3–5.5)	0.6025
Sex	Female	114 (94.2)	7 (5.8)	0.8666
	Male	75 (93.7)	5 (6.2)	

**Table 5:** Odds ratios (OR) of factors influencing WNV infection in dogs in Bulgaria.

Variable		OR	95% CI	*p* Value
Region	Northwestern	3.3896	1.2249 to 9.3800	0.0187
	North-central	-		
	Northeastern	2.5714	0.8054 to 8.2098	0.1108
District	Vratsa	1.0324	0.4199 to 2.5385	0.9446
	Pleven	-		
	Ruse	0.2982	0.1030 to 0.8639	0.0258
	Silistra	0.7669	0.3186 to 1.8458	0.5537
Age *	<2 y			
	≥2 y	3.0472	1.2101 to 7.6734	0.0180

* Age categories: <2 years; ≥2 years (AUC = 0.609; *p* = 0.0055).

## Data Availability

The data presented in this study are available within the article.

## Supplementary Materials

The following supporting information can be downloaded at: https://www.mdpi.com/article/10.3390/vetsci12040373/s1, Table S1: Distribution of the tested samples by provinces, sex and age.
