# Supplementary material for: First Serologic Evidence of West Nile Virus and Usutu Virus Circulation Among Dogs in the Bulgarian Danube Region and Analysis of Some Risk Factors
Source: Vet Sci. 2025 Apr 16;12(4):373. doi: 10.3390/vetsci12040373 (PMC12031095; doi:10.3390/vetsci12040373)
Supplement: Supplementary file 1 [file vetsci-12-00373-s001.zip › vetsci-3561519-supplementary.pdf]

Table 1. Distribution of the tested samples by districts, sex and age.

District	N samples	N males	N females	Age, years	% samples
Vratsa	36	16	20	1–11	17,9
Pleven	70	26	44	6m–8	34,8
Ruse	50	19	31	1–13	24,9
Silistra	45	19	26	1–13	22,4
**Total**	**201**	**80**	**121**	**6m-13**	**100**
